# Real-life use of vasopressors and inotropes in cardiogenic shock—observation is necessarily ‘theory-laden’

**DOI:** 10.1186/s13054-016-1473-4

**Published:** 2016-09-26

**Authors:** Samiran Ray, Mirjana Cvetkovic, Daniel H. Lutman, Nazima Pathan, Padmanabhan Ramnarayan, David P. Inwald, Mark J. Peters

**Affiliations:** 1 Children’s Acute Transport Service, Great Ormond Street Hospital NHS Trust, London, WC1N 3JH UK; 2 University Hospitals of Leicester NHS Trusts, Leicester, UK; 3 Addenbrookes Hospital, Cambridge University Hospitals NHS Trust, Cambridge, UK; 4 Imperial College Healthcare NHS Trust, London, UK; 5 University College London Institute of Child Health, 30 Guildford Street, London, UK

We commend the attempt of Tarvasmäki et al. [1] to identify the mortality risk associated with individual vaso-active agents used in cardiogenic shock. However, despite their rigorous statistical analysis, we recommend caution in interpreting these results. Propensity score matching accounts for prior bias in the choice of vaso-active agents, but this choice of agent is often deeply engrained in individual clinician dogma which even propensity score matching may not uncover.

We recently conducted a similar retrospective analysis of vaso-active agents used in children with sepsis during stabilisation and transport of children by our paediatric intensive care transport service. Cold shock is common in sepsis in children, and the choice of vaso-active agent depends on the balance of inotropy or vasoconstriction needed. During transport, haemodynamic assessment is limited—choices of vaso-active agent used are made on limited data. Over a 7-year period (2005–2011), 364/633 (57.5 %) children were started at least on one vaso-active agent prior to intensive care unit admission. Epinephrine was associated with a crude mortality of 3.84 (95 % CI 2.5–5.91) compared to 1.8 (95 % CI 1.17–2.76) and 1.39 (95 % CI 0.91–2.1) for norepinephrine and dopamine, respectively. However, when standardised for risk using the PIM (Paediatric Index of Mortality) score, the standardised mortality ratio for epinephrine was 1.30 (95 % CI 0.98–1.63). When propensity score matching was used to account for choice of agent (calculated using age, weight, fluids administered, need for prior CPR, other agent use), no difference was seen between those who did and did not receive epinephrine (*p* value = 0.09). Yet Ventura et al. demonstrated, via a randomised controlled trial, that dopamine was associated with an increased mortality in children with sepsis compared to the use of epinephrine (OR 6.5, 95 % CI 1.1–37.8) [2]!

These contradictory findings are likely be a reflection of how different drugs are used—as the degree of statistical adjustment for confounding was improved (from crude to standardised mortality to propensity score matching), the initial mortality association with epinephrine use disappeared. When the use of drugs was protocolised in randomised controlled trial circumstances the association was reversed, with epinephrine showing a mortality benefit over dopamine. It is likely that more important than the choice of drug may be the use of the right amount of the right drug at the right time [3]. We join the authors to call for further randomised controlled trials to determine an evidence-based approach to vaso-active agent use.

## Additional file

Additional file 1: Contingency table with data used to calculate crude odd ratios of death for each vasoactive agent. (XLSX 11 kb)

## Abbreviations

OR: Odds ratio
CI: Confidence interval
